# Fetal abdominal overgrowth is already present at 20–24 gestational weeks prior to diagnosis of gestational diabetes mellitus

**DOI:** 10.1038/s41598-021-03145-7

**Published:** 2021-12-10

**Authors:** Wonjin Kim, Soo Kyung Park, Yoo Lee Kim

**Affiliations:** 1 Division of Endocrinology and Metabolism, Department of Internal Medicine, CHA Gangnam Medical Center, CHA University School of Medicine, 566, Nonhyeon-ro, Gangnam-gu, Seoul, 06135 Republic of Korea; 2 Yonsei University College of Medicine, Seoul, Republic of Korea; 3 Department of Biostatistics and Data Science, University of Texas Health Science Center at Houston, Houston, TX 77030 USA

**Keywords:** Endocrinology, Endocrine system and metabolic diseases

## Abstract

Fetal abdominal obesity (FAO) was detected at the time of gestational diabetes mellitus (GDM) diagnosis at 24–28 gestational weeks (GW) in older (≥ 35 years) and/or obese (≥ body mass index 25 kg/m^2^) women and persisted until delivery. We investigated whether FAO is already present at 20–24 GW. Medical records of 7820 singleton pregnancy including 384 GDM were reviewed. Fetal abdominal overgrowth was assessed by the fetal abdominal overgrowth ratios (FAORs) of the ultrasonographically estimated gestational age (GA) of abdominal circumference per actual GA by the last menstruation period, biparietal diameter or femur length, respectively. FAO was defined as FAOR ≥ 90th percentile. FAORs measured at 20–24 GW in older and/or obese but not in young and non-obese GDM subjects were significantly higher than those in NGT subjects. Relative to NGT subjects without FAO at 20–24 GW, odds ratios for exhibiting FAO at GDM diagnosis and large for gestational age in GDM with FAO at 20–24 GW were 10.15 and 5.57, and their primary cesarean delivery rate was significantly higher than those in GDM without FAO (44% vs. 29%). Earlier diagnosis and active interventions of GDM well before 20–24 GW might be necessary to prevent FAO in the older and/or obese women.

## Introduction

Childhood obesity is increasing worldwide with the incidence of type 2 diabetes in adolescents^1–3^. Gestational diabetes mellitus (GDM) is a risk factor for childhood obesity in offspring. Although management of GDM reduced the rate of large for gestational age (LGA) and macrosomia, it did not fully normalize neonatal outcomes, including the rates of neonatal hypoglycemia, raised cord blood C-peptide levels^4,5^. Furthermore, no reduction in childhood obesity or metabolic dysfunction in the offspring of treated GDM subjects has been reported, even though only female offspring showed significantly lower fasting glucose^6,7^. This controversial situation raises the question whether the onset of fetal overgrowth among women subsequently diagnosed with GDM might occur well before the diagnosis of GDM at ≤ 28 gestational weeks (GW). Sovio et al. reported that diagnosis of GDM was preceded by fetal abdominal obesity (FAO) between 20–28 GW, and its effect on FAO was additive with the effect of maternal obesity^8^. A prospective cohort study that investigates fetal growth by ultrasonography revealed that fetus of GDM subjects started to show larger estimated fetal weight (EFW) at 20 GW and became statistically significant at 28 GW and showed smaller HC (head circumference)/AC (abdominal circumference) ratio in the later pregnancy^9^.

Venkataraman et al. observed a ‘thin but fat’ phenotype, that is disproportionate increase in adiposity despite smaller or similar lean body mass, in the fetus of GDM mother even at 20 GW, predating the biochemical diagnosis of GDM^10^. The link between maternal glucose level and neonatal adiposity has been confirmed, and the relationship was suggested to be mediated by fetal insulin production^11–13^. Thus, FAO is mainly caused by fetal hyperinsulinemia together with excess maternal fuel. As fetal hyperinsulinemia as early as 14–16 GW was observed in the fetus of mother with diabetes, much earlier diagnosis and intervention than the current diagnosis of GDM at 24–28 GW might be necessary to prevent fetal obesity and macrosomia^13^.

According to Brunetti et al.’s Italian study^14^, early GDM screening at 16–18 GW in high risk women^15^ reduced AC and EFW measured at 20.6 GW and led to reduced infant birth weight, and these parameters were all comparable with those in low and medium risk GDM and NGT subject screened late. Also, AC and EFW percentile in medium and low risk group were significantly higher than those in NGT group but there was no significant difference in birth weight percentile^16^.

We previously reported that FAO is already present at diagnosis of GDM at 24–28 GW in the older (≥ 35 years) and/or obese (BMI ≥ 25 kg/m^2^) GDM subjects but not in young and non-obese GDM subjects^17^. These findings suggest that while screening and diagnosis of GDM at 24–28 GW is not late for young (< 35 years) and non-obese (BMI < 25 kg/m^2^) women, much earlier diagnosis and intervention might be necessary in the older and/or obese women to prevent FAO resulting from fetal hyperinsulinemia.

In this study, we investigated whether FAO is already present at 20–24 GW in GDM mother in order to determine the appropriate time for early diagnosis and treatment of GDM to prevent FAO.

## Results

### Clinical and biochemical characteristics and pregnancy outcomes of the normal glucose tolerance and GDM subjects according to maternal age and pre-pregnancy BMI

As we previously reported^18^, maternal age and pre-pregnancy BMI were significantly higher in patients with GDM, but weight gain until diagnosis of GDM was not higher as compared with normal glucose tolerance (NGT) subjects.

HbA1c measured at diagnosis was significantly higher in the obese group 2 and 4 GDM than in the non-obese group 1 and 3 GDM. There was a significantly higher frequency of insulin treatment during pregnancy in the obese group 2 and 4 compared with the young and non-obese group 1 GDM (17.4% and 15.7% vs. 8.2%, *p* < 0.05).

The prevalence of LGA at birth (12.7% vs. 5.1%, *p* < 0.05), macrosomia (4.5% vs. 1.9%, *p* < 0.05), and primary cesarean delivery rates (30.2% vs. 22.5%, *p* < 0.05) were significantly higher in the GDM patients than in the NGT subjects. In the subgroup analysis, as compared with NGT subjects, all GDM subgroups had a higher prevalence of LGA at birth and macrosomia, but young and non-obese group 1 GDM did not (Table 1, published in Ref.^15^)^18^.

**Table 1:** Clinical and biochemical characteristics and pregnancy outcomes of NGT and GDM subjects according to maternal age and pre-pregnancy BMI.

	NGT (n = 6721)	Total GDM (n = 378)	GDM			
			Group 1 (n = 144)	Group 2 (n = 22)	Group 3 (n = 162)	Group 4 (n = 50)
**Clinical and biochemical**						
Age (years)	33.1 ± 3.8	35.3 ± 4.0^a^	31.7 ± 1.7^a^	31.4 ± 2.2	38.0 ± 2.6^a,b,c^	38.6 ± 2.6^a,b,c^
Pre-pregnancy BMI (kg/m^2^)	20.6 ± 2.6	22.3 ± 3.5^a^	20.6 ± 2.0	28.0 ± 2.8^a,b^	21.3 ± 2.0^a,c^	28.0 ± 2.4^a,b,d^
Weight gain (kg)						
Pre-pregnancy—at diagnosis	7.6 ± 3.2	7.8 ± 3.6	8.3 ± 3.7	6.7 ± 4.3^a^	8.0 ± 3.4	6.9 ± 3.4^a^
Pre-pregnancy—near term	13.0 ± 4.1	11.5 ± 4.6^a^	12.2 ± 4.5	9.3 ± 6.4^a,b^	11.7 ± 4.1^a^	9.5 ± 4.8^a,b^
50-g GCT (mmol/L)	6.2 ± 1.2	9.2 ± 1.4^a^	9.0 ± 1.1^a^	9.6 ± 1.4^a^	9.1 ± 1.1^a^	9.6 ± 2.4^a^
Fasting plasma glucose^f^ (mmol/L)	4.5 ± 0.4	5.0 ± 0.8^a^	4.8 ± 0.6^a^	5.5 ± 0.9^a,b^	4.9 ± 0.6^a,c^	5.3 ± 1.5^a,b,d^
HbA1c at diagnosis^f^ (%)	5.0 ± 0.3	5.3 ± 0.5^a^	5.2 ± 0.3^a^	5.6 ± 0.6^a,b^	5.2 ± 0.3^a,c^	5.6 ± 1.0^a,b,d^
HbA1c at diagnosis^f^ (%) (mmol/mol)	31.3 ± 3.5	34.4 ± 6.0	33.4 ± 4.1	37.6 ± 6.9	33.9 ± 4.1	38.6 ± 10.8
Fasting insulin^f^ (pmol/L)	54.9 ± 29.2	68.8 ± 34.7^a^	65.3 ± 34.8	111.8 ± 35.4^a,b^	60.4 ± 26.4^c^	88.2 ± 32.6^a,b,c,d^
Insulin treatment (%)	–	10.4	8.2	17.4^b^	9.8	15.7^b^
**Pregnancy outcomes**						
Primipara (%)	70.2	66.7	77.1	81.8	59.9^a^	52.0^a^
Cesarean delivery (%)	33.9	46.8^a^	30.6	40.9	58.6^a^	58.0^a^
Primary cesarean delivery (%)	22.5	30.2^a^	22.9	36.4	35.8^a^	30.0
Infant birth weight (g)	3197 ± 421	3226 ± 510	3157 ± 487	3303 ± 677^b^	3262 ± 452^a,b^	3265 ± 654
GA at delivery (weeks)	39.0 ± 1.5	38.4 ± 1.8^a^	38.5 ± 1.8^a^	38.2 ± 2.2^a^	38.5 ± 1.5^a^	37.8 ± 2.4^a^
LGA (%)	5.1	12.7^a^	7.6	18.2^a^	14.2^a^	20.0^a^
Macrosomia^e^ (%)	1.9	4.5^a^	3.5	4.5	4.3^a^	8.0^a^

*NGT* normal glucose tolerance, *GDM* gestational diabetes mellitus, *BMI* body mass index, *GCT* glucose challenge test, *HbA1c* glycosylated hemoglobin, *GA* gestational age, *LGA* large for gestational age; GDM group 1 (age < 35 years and BMI < 25 kg/m^2^); group 2 (age < 35 years and BMI ≥ 25 kg/m^2^); group 3 (age ≥ 35 years and BMI < 25 kg/m^2^); group 4 (age ≥ 35 years and BMI ≥ 25 kg/m^2^).

^a^p < 0.05, compared with NGT, ^b^p < 0.05, compared with GDM group 1, ^c^p < 0.05, compared with GDM group 2, ^d^p < 0.05, compared with GDM group 3, ^e^Infant birth weight ≥ 4 kg, ^f^Column Ns for the variable are different from the Ns presented in the table.

### Results of fetal biometry and fetal abdominal overgrowth ratios (FAORs) in the NGT, GDM, and GDM subgroups

While actual gestational age (GA) determined by last menstrual period (LMP) and estimated GA of biparietal diameter (BPD) and femur length (FL), and EFW by ultrasonography done at 20–24 GW, 4 weeks prior to screening with 50-g glucose tolerance test (GCT), were similar among study groups, estimated GA of AC was significantly higher in total GDM and older and non-obese group 3 GDM subjects than those in NGT subjects (Table 2).

**Table 2:** Results of fetal biometry and FAORs measured at 20–24 GW in the subjects subsequently diagnosed with NGT and GDM.

	NGT (n = 6639)	Total GDM (n = 357)	GDM			
			Group 1 (n = 135)	Group 2 (n = 20)	Group 3 (n = 154)	Group 4 (n = 48)
**Fetal biometry**						
GA-LMP^a^	22.1 ± 0.9	22.1 ± 0.9	22.1 ± 0.9	21.9 ± 1.0	22.1 ± 0.9	22.0 ± 0.8
GA-AC (week)	22.7 ± 1.1	22.9 ± 1.1^b^	22.8 ± 1.1	23.0 ± 1.3	22.9 ± 1.2^b^	22.8 ± 1.0
GA-BPD (week)	22.4 ± 1.2	22.4 ± 1.1	22.4 ± 1.2	22.1 ± 1.5	22.5 ± 1.1	22.4 ± 0.9
GA-FL (week)	22.2 ± 1.1	22.2 ± 1.1	22.2 ± 1.2	22.1 ± 1.3	22.2 ± 1.1	22.2 ± 1.0
EFW (g)	515.5 ± 93.0	519.7 ± 91.3	515.3 ± 89.9	514.8 ± 120.8	524.9 ± 92.3	517.4 ± 79.4
**FAOR**						
GA-AC/GA-LMP^a^	1.027 ± 0.037	1.034 ± 0.038^c^	1.029 ± 0.035	1.046 ± 0.027^b^	1.036 ± 0.041^b^	1.037 ± 0.037^b^
GA-AC/GA-BPD	1.013 ± 0.043	1.019 ± 0.043^b^	1.017 ± 0.044	1.039 ± 0.038^b^	1.019 ± 0.042	1.019 ± 0.040
GA-AC/GA-FL	1.023 ± 0.040	1.030 ± 0.043^c^	1.026 ± 0.044	1.040 ± 0.040	1.032 ± 0.043^b^	1.030 ± 0.040

*GW* gestational weeks, *FAOR* fetal abdominal overgrowth ratio, *NGT* normal glucose tolerance, *GDM* gestational diabetes mellitus, *BMI* body mass index, *GA-LMP* gestational age by last menstruation period, *GA-AC* estimated gestational age by abdominal circumference, *GA-BPD* estimated gestational age by biparietal diameter, *GA-FL* estimated gestational age by femur length, *EFW* estimated fetal weight; GDM group 1 (age < 35 years and BMI < 25 kg/m^2^); group 2 (age < 35 years and BMI ≥ 25 kg/m^2^); group 3 (age ≥ 35 years and BMI < 25 kg/m^2^); group 4 (age ≥ 35 years and BMI ≥ 25 kg/m^2^).

^a^Gestational age by LMP at 20–24 GW, ^b^p < 0.05, compared with NGT, ^c^p < 0.001, compared with NGT.

All fetal abdominal overgrowth ratios (FAORs) of total GDM subjects were significantly higher than those of NGT subjects. In subgroup analysis, FAOR of GA-AC/GA-LMP in group 2, 3, and 4 GDM, GA-AC/GA-BPD in group 2 GDM, and GA-AC/GA-FL in group 3 GDM were significantly higher than those in NGT subjects. However, all FAORs of young and non-obese group 1 GDM subjects were not significantly different from those of NGT subjects (Table 2).

According to investigation of the birth characteristics of those without fetal biometry data, there was no significant difference among GDM subjects but NGT subjects without fetal biometry dada showed lower rate of LGA at birth and primary cesarean delivery. Thus, we suggest, especially for GDM subjects, the current pregnancy outcome data presented in Table 1 were not biased by the availability of fetal biometry data (Supplementary Table S1).

### Odds ratios for FAO in NGT, GDM and GDM subgroups

Relative to NGT subjects, odds ratio for FAO indicated by GA-AC/GA-LMP ≥ 90th percentile and GA-AC/GA-FL ≥ 90th percentile in total GDM subjects was 1.496 (95% CI 1.097–2.038) and 1.589 (1.171–2.156), respectively (Supplementary Table S2).

In group 1 GDM subjects, odd ratios for FAO by all FAORs were not significantly higher than those in NGT group. On the other hand, odds ratios for FAO were 3.915 (1.500–10.233) in group 2 GDM, as indicated by FAOR of GA-AC/GA-BPD and 2.151 (1.037–4461) in group 4 GDM by GA-AC/GA-FL. Odds ratios for FAO in group 3 GDM was 2.041 (1.244–3.098) by FAOR of GA-AC/GA-LMP and 1.637 (1.043–2.568) by GA-AC/GA-FL (Supplementary Table S2).

### FAORs and odds ratios for FAO according to GW in NGT and GDM subjects

The FAOR of GA-AC/GA-LMP in GDM subjects was not significantly higher than those of NGT at 20 GW, but those became significantly higher at 21 and 22 GW and this tendency maintained until 23 GW (Table 3). Also, odds ratio for FAO in the GDM subjects was significantly increased relative to that of NGT subjects at 21 GW. But EFW of GDM subjects tended to higher than EFT of NGT subjects without significance (Supplementary Table S3).

**Table 3:** FAORs and EFW according to measured GW in the subjects subsequently diagnosed with NGT and GDM.

	NGT^a^	GDM^b^	p-value
**GA-AC/GA-LMP**			
20–21 GW	1.035 ± 0.039	1.042 ± 0.036	0.3688
21–22 GW	1.030 ± 0.036	1.038 ± 0.037	0.0153
22–23 GW	1.026 ± 0.037	1.034 ± 0.039	0.0093
23–24 GW	1.018 ± 0.037	1.022 ± 0.036	0.4567
**GA-AC/GA-BPD**			
20–21 GW	1.017 ± 0.043	1.020 ± 0.041	0.7431
21–22 GW	1.014 ± 0.042	1.021 ± 0.045	0.0717
22–23 GW	1.012 ± 0.043	1.019 ± 0.042	0.0518
23–24 GW	1.010 ± 0.043	1.017 ± 0.042	0.9459
**GA-AC/GA-FL**			
20–21 GW	1.029 ± 0.039	1.030 ± 0.034	0.8217
21–22 GW	1.027 ± 0.040	1.035 ± 0.042	0.0252
22–23 GW	1.022 ± 0.040	1.030 ± 0.045	0.0148
23–24 GW	1.015 ± 0.041	1.023 ± 0.044	0.2025
**EFW (g)**			
20–21 GW	396.2 ± 50.8	406.3 ± 62.5	0.3138
21–22 GW	460.3 ± 55.5	469.7 ± 50.5	0.0649
22–23 GW	537.5 ± 63.3	544.9 ± 60.6	0.1643
23–24 GW	620.2 ± 70.5	615.3 ± 61.1	0.6313

*FAOR* fetal abdominal overgrowth ratio, *EFW* estimated fetal weight, *NGT* normal glucose tolerance, *GDM* gestational diabetes mellitus, *GW* gestational week, *GA-AC* estimated gestational age by abdominal circumference, *GA-LMP* gestational age by last menstruation period, *GA-BPD* estimated gestational age by biparietal diameter, *GA-FL* estimated gestational age by femur length.

^a^Number of NGT subjects having available data for FAORs and EFW at GW 20–21, 21–22, 22–23, and 23–24 were 486, 2344, 2688, and 951, respectively. ^b^Number of GDM subjects having available data for FAORs and EFW at GW 20–21, 21–22, 22–23, and 23–24 were 28, 124, 148, and 48, respectively.

### Clinical characteristics and pregnancy outcomes of GDM and NGT subjects based on presence or absence of FAO at 20–24 GW

Comparing with the FAO (−) groups, maternal age of the FAO (+) groups was significantly higher in both NGT and GDM subjects, but pre-pregnancy BMI and weight gain until diagnosis of GDM of the FAO (+) groups were significantly higher in NGT but not in GDM subjects.

While fasting plasma glucose on 100-g oral glucose tolerance test (OGTT), HbA1c and HOMA-β (homeostatic model assessment for insulin resistance) of the FAO (+) groups were not significantly different from those of the FAO (−) groups in both NGT and GDM subjects, HOMA-IR (homeostatic model assessment for insulin secretion) of the FAO (+) group was significantly lower than those of the FAO (−) group in GDM but not in NGT subjects. With HOMA-IR, GDM subjects showed significantly higher values than NGT subjects only in FAO (−) groups (2.3 ± 1.2 vs. 1.6 ± 0.9, p < 0.05) but not in FAO (+) groups (1.9 ± 0.9 vs. 1.4 ± 0.7, p > 0.05). With HOMA-β, GDM subjects showed lower values than NGT subjects in FAO (+) groups (153.6 ± 142.6 vs. 213.2 ± 167.5, p > 0.05) without significance whereas those in FAO (−) groups were comparable with each other (157.2 ± 89.1 vs. 172.1 ± 92.3, p > 0.05).

The frequency of FAO at diagnosis of GDM was significantly higher in the FAO (+) group than in the FAO (−) group in both NGT and GDM subjects. While male sex and primary cesarean delivery rate of the FAO (+) groups were significantly higher than those of the FAO (−) groups in both NGT and GDM subjects, the frequency of primipara, LGA at birth, and macrosomia of the FAO (+) groups were significantly higher than those of the FAO (−) groups in NGT but not in GDM subjects (Table 4).

**Table 4:** Clinical and biochemical characteristics and pregnancy outcomes by presence or absence of FAO at 20–24 GW in the subjects subsequently diagnosed with NGT and GDM.

	NGT (n = 6639)		GDM (n = 357)	
	FAO (−) (n = 5987)	FAO (+) (n = 652)	FAO (−) (n = 307)	FAO (+) (n = 50)
**Clinical and biochemical**				
Age (years)	33.1 ± 3.7	34.0 ± 3.9^a^	35.1 ± 3.9	36.6 ± 4.0^a^
Pre-pregnancy BMI (kg/m^2^)	20.6 ± 2.5	20.8 ± 2.7^a^	22.4 ± 3.5	22.0 ± 2.9
Weight gain (kg)				
Pre-pregnancy—at diagnosis	7.5 ± 3.3	8.0 ± 3.1^a^	7.8 ± 3.5	8.3 ± 4.0
HbA1c at diagnosis (%)^b^	5.0 ± 0.3	5.0 ± 0.2	5.3 ± 0.5	5.2 ± 0.4
HbA1c at diagnosis (mmol/mol)^b^	31.3 ± 3.5	31.3 ± 3.0	34.4 ± 6.0	33.9 ± 5.0
FPG on 100-g OGTT (mg/dL)^b^	80.2 ± 6.5	80.3 ± 5.7	89.7 ± 15.1	87.7 ± 10.3
FPG on 100-g OGTT (mmol/L)^b^	4.5 ± 0.4	4.5 ± 0.3	5.0 ± 0.8	4.9 ± 0.6
HOMA-IR^b^	1.6 ± 0.9	1.4 ± 0.7	2.3 ± 1.2	1.9 ± 0.9^a^
HOMA-β^b^	172.1 ± 92.3	213.2 ± 167.5	157.2 ± 89.1	153.6 ± 142.6
FAO (+) at diagnosis (%)	5.6	20.4^a^	11.1	34.0^a^
**Pregnancy outcomes**				
Primipara (%)	66.9	71.2^a^	65.5	74.0
Male sex of infant (%)	50.3	73.6^a^	52.4	80.0^a^
LGA (%)	4.0	12.3^a^	11.7	20.0
Macrosomia (%)	1.6	4.1^a^	4.6	4.0
Cesarean delivery (%)	32.2	37.1^a^	45.9	56.0
Primary cesarean delivery (%)	21.5	26.1^a^	29.0	44^a^
Male sex of infant (%)	50.3	73.6^a^	52.4	80.0^a^
LGA (%)	4.0	12.3^a^	11.7	20.0
Macrosomia (%)	1.6	4.1^a^	4.6	4.0

*FAO* fetal abdominal obesity, *GW* gestational weeks, *NGT* normal glucose tolerance, *GDM* gestational diabetes mellitus, *BMI* body mass index, *HbA1c* glycosylated hemoglobin, *FPG* fasting plasma glucose, *OGTT* oral glucose tolerance test, *HOMA-IR* homeostatic model assessment for insulin resistance, *HOMA-β* homeostatic model assessment for insulin secretion, *LGA* large for gestational age.

^a^p < 0.05 compared to FAO (–), ^b^Column Ns for the variable are different from the Ns presented in the table.

### Odds ratios for FAO at the time of GDM diagnosis, being LGA at birth, and macrosomia

Relative to the FAO (−) NGT subjects at 20–24 GW, FAO (+) NGT subjects revealed odds ratios for exhibiting FAO at diagnosis of GDM, LGA at birth, and macrosomia as 4.56 (95% CI 3.63–5.74), 3.5 (2.68–4.59), and 2.68 (1.73–4.14), respectively.

While FAO (−) GDM subjects at 20–24 GW showed odds ratios for exhibiting FAO at diagnosis of GDM 2.03 (1.39–2.97), LGA at birth, 3.10 (2.14–4.51), and macrosomia, 2.96 (1.67–5.26) relative to FAO (−) NGT subjects, FAO (+) GDM subjects at 20–24 GW showed markedly high odds ratios for FAO at diagnosis of GDM 10.15 and LGA at birth 5.57, but not the ratio for macrosomia (Table 5).

**Table 5:** Odds ratios of FAO at the time of diagnosis of GDM, LGA at birth, and macrosomia by NGT and GDM subjects and by presence or absence of FAO at 20–24 GW.

	Odds ratio (95% CI)		
	FAO at diagnosis (+)	LGA at birth	Macrosomia
**NGT**			
FAO^a^ at 20–24 GW (−)	1 (ref.)	1 (ref.)	1 (ref.)
FAO at 20–24 GW (+)	4.56^b^ (3.63, 5.74)	3.50^b^ (2.68, 4.59)	2.68^b^ (1.73,4.14)
**GDM**			
FAO at 20–24 GW (−)	2.03^b^ (1.39, 2.97)	3.10^b^ (2.14, 4.51)	2.96^b^ (1.67, 5.26)
FAO at 20–24 GW (+)	10.15^b^ (5.27, 19.57)	5.57^b^ (2.75, 11.29)	2.58 (0.62, 10.79)

*FAO* fetal abdominal obesity, *GDM* gestational diabetes mellitus, *LGA* large for gestational age, *GW* gestational weeks, *NGT* normal glucose tolerance.

^a^Fetal abdominal obesity defined as fetal abdominal overgrowth ratio ≥ 90th, ^b^p < 0.05 compared with NGT FAO (−).

### Correlation of FAORs measured at 20–24 GW with FAORs measured at the time of GDM diagnosis

All FAORs measured at 20–24 GW showed significant correlation with those subsequently measured at the time of GDM diagnosis (Supplementary Table S4).

## Discussion

The association between GDM and increased fetal adiposity has been known to be limited to late pregnancy^19–22^ and appropriate management of GDM initiated even at ≥ 30 GW^23^ was reported to reduce the risks of GDM complications such as fetal overgrowth, shoulder dystocia and cesarean delivery^4,5^. So, the universal GDM screening of pregnant women at 24–28 GW is now recommended by many professional societies^24–27^. However, childhood obesity or metabolic dysfunction in the offspring of mild GDM subjects was not reduced by treatment of GDM^6,7^. This suggests that current management to reduce LGA and macrosomia is not sufficient for the prevention of long-term complication of GDM in the offspring.

FAO might serve as an early indicator of GDM and GDM complication. Fetal abdominal overgrowth was observed earlier than usual time of GDM diagnosis^10^ and even in the fetus with appropriate weight for gestational age^28^. Moreover, FAO might also be a significant risk factor for early childhood and later in life obesity^28,29^.

According to our previous studies, GDM diagnosed at 24–28 GW has already affected FAO in the older (≥ 35 years) and/or obese (BMI ≥ 25 kg/m^2^) women but not in the young and non-obese women^17^ and FAO persisted until delivery despite treatment of GDM^18^. The prevalence of near-term FAO leading to significantly higher infant birth weight and cesarean section rate were threefold higher in GDM with FAO than without FAO at 24–28 GW^18^.

In the present study, FAO was already observed at 20–24 GW, well predating the time of GDM diagnosis at 24–28 GW, in the older and/or obese but not in the young and non-obese women. Furthermore, the odds ratios for exhibiting FAO at diagnosis of GDM and LGA at birth were tenfold and fivefold higher, respectively, in the GDM subjects with FAO than NGT subjects without FAO at 20–24 GW.

Sovio et al. reported that fetal abdominal overgrowth was not observed at 20 GW in the women who subsequently diagnosed with GDM. However, obese women already had an increased risk of fetal abdominal overgrowth in fetal biometry at that time and were at higher risk if later diagnosed with GDM^8^.

Overall, these findings suggest that while the current universal GDM screening and treatment initiated at 24–28 GW might be appropriate for the young and non-obese women, much earlier GDM diagnosis before the onset of FAO and careful management would be necessary to prevent the development of FAO in the high risk older and/or obese women.

Determining the optimal time frame for GDM screening and initiation of treatment before the onset of FAO might be a clinically important issue to improve the maternal and newborn outcomes. Several studies reported that earlier recognition of women at risk for the development of GDM and other adverse pregnancy outcomes might benefit from earlier detection and intervention^15,30,31^. However, in a study that GDM subjects were stratified by GA at initiation of treatment from 24 to 30 GW, earlier initiation of GDM treatment was not associated with favorable pregnancy outcomes^23^. According to Sweeting et al., pregnancy outcomes of women who was diagnosed with GDM before 12 GW and had higher HbA1c at diagnosis was poor and similar to those of pre-existing diabetes despite early testing and best practice treatment. But GDM subjects diagnosed at 12–23 GW and after 24 GW showed similar frequency of LGA at birth and macrosomia^32^. Considering that FAO was already present at 20–24 GW and frequency of LGA is significantly higher in the GDM subjects with FAO than those without FAO in our study, the pregnancy outcome of the earlier diagnosed GDM subjects at 12–23 GW can be interpreted as favorable results of early diagnosis, while initiation of treatment later than 24 GW was thought to be too late to expect favorable outcome. For the GDM subjects diagnosed before 12 GW, it is thought that pre-pregnancy screening for type 2 diabetes mellitus should have been performed.

According to meta-analysis by Immanuel et al.^33^, relative risk for perinatal mortality, neonatal hypoglycemia, and insulin use in early-onset (< 24 GW) GDM women were 3.58, 1.61, and 1.71, respectively compared to late-onset (24–28 GW) GDM women. In the pilot randomized controlled trial (RCT) with pregnant women who had GDM risk factors and took an OGTT earlier than 20 GW, pregnancy outcomes were compared between women receiving immediate or deferred treatment after booking^34^. Early treatment of booking GDM subjects reduced frequency of neonatal macrosomia instead of increasing the frequency of SGA infants. Therefore, a full multicenter RCT for 4000 pregnant women (< 20 GW) at risk of overt diabetes in pregnancy is underway under the name of TOBOGM (treatment of booking gestational diabetes mellitus) study^35^.

In our study, FAOR of GDM subject began to deviate significantly from those of NGT subjects at 21 GW and odds ratio for FAO in GDM subjects was significantly increased at 21 GW. In concordance with our data, Sovio et al. reported the development of FAO between 20 and 28 GW in the GDM subjects^8^. Also, fetus of GDM subjects were reported to begin to have higher EFW than NGT fetus at 20 GW, the difference reached statistical significance at 28 GW, and showed abdominal overgrowth compared to head growth at 29 GW^9^. But FAO was observed even at 20 GW, far predating the biochemical diagnosis of GDM, in the South African study of Venkataraman et al.^10^.

Collectively, these findings also suggest that much earlier diagnosis of GDM well before 20 GW would be necessary to prevent the development of FAO and subsequent progress to LGA infants and childhood obesity in the high risk older and/or obese women.

It is well known that both the fetal glucose homeostasis and the trajectory of intrauterine growth are consequences of intricate connections between the fetal endocrine system and placental function, which is affected by uterine blood flow and maternal health and nutrition, especially in elderly primiparas^36^. Nevertheless, maternal hyperglycemia was the strongest predictor of FAO in GDM subjects^12,28,37^ because fetal energy requirement for metabolism and growth is met mainly by glucose from mother^38^. In the subgroup analysis of this study, GDM subjects with FAO at 20–24 GW was not more obese but older than the subjects without FAO at 20–24 GW. In addition, while HOMA-IR of GDM with FAO at 20–24 GW was not significantly higher than those of NGT with FAO, HOMA-β was lower than NGT without significance. This finding suggests that maternal metabolic abnormality of GDM mother with FAO at 20–24 GW is primarily reduced insulin secretion due to maternal old age, rather than markedly increased insulin resistance. As a result, maternal hyperglycemia occurred in early pregnancy and fetal hyperinsulinemia with FAO was developed subsequently at 20–24 GW in the high risk older and/or obese GDM women. Furthermore, it is thought that hyperinsulinemic fetuses may have lowered maternal glucose levels through exaggerated glucose steal^39^.

Our findings that FAO leading to adverse pregnancy outcome is associated with decreased insulin secretion rather than insulin resistance is different from the European data that GDM women with high insulin resistance showed poor pregnancy outcomes^40^. Recently, metabolic phenotypes of early GDM based on insulin resistance and secretion and their association with adverse pregnancy outcomes was reported^41^. Compared with NGT women, GDM women with high insulin resistance had a greater risk of having LGA and cesarean delivery while GDM women with lower insulin secretion or both abnormalities had comparable pregnancy outcome with those in NGT women. These findings are explained by ethnic difference that Asians have higher insulin sensitivity and lower insulin response than Caucasians^42^. In addition, decreased insulin secretion appears to be a major factor in the development of type 2 diabetes in Asian population^43^.

It is of note that fetal fat deposition begins at 14 GW coinciding with the onset time of early fetal hyperinsulinemia^13^. In the present study, FAO detected at 20–24 GW increased the odds for exhibiting FAO at GDM diagnosis 5 folds in GDM subjects (10.15 vs. 2.03, Table 5), but odds ratio for LGA at birth and macrosomia were elevated less than 2 folds with the management of GDM in comparison with GDM subjects without FAO at 20–24 GW. In concordance with our data, there is a report that while birth weight was normalized with tight glycemic control in later pregnancy, but already developed fetal hyperinsulinemia resulting in persistent FAO was difficult to normalize^28^. Therefore, to further improve pregnancy outcomes related to fetal hyperinsulinemia and the ensuing FAO in the high risk older and/or obese GDM as well as in pre-existing diabetes, pre-pregnancy planning and better means to optimize metabolic control early in pregnancy would be necessary. However, in young and non-obese women who are not at high risk for fetal overgrowth and women without family history of diabetes and history of previous GDM and delivery of macrosomic baby, screening for GDM and initiation of medical nutrition therapy at 24–28 GW might be effective to prevent adverse birth outcomes.

Normalization of fetal hyperinsulinemia which is related with maternal hyperglycemia is most important for the prevention of fetal abdominal overgrowth. Initiation of insulin treatment in GDM subjects according to amniotic fluid insulin concentration markedly reduced the rate of elevated cord blood c-peptide levels in neonates^44^. Measurement of insulin in amniotic fluid or cord blood is not easy for detection of fetal hyperinsulinemia. If the association between FAORs and insulin or c-peptide levels in cord blood or amniotic fluid is defined through investigation, FAORs which we measured could be used as a surrogate marker for fetal hyperinsulinemia.

The limitations of the present study include the single center, retrospective, and uncontrolled observational study design. So, number of subjects scanned by ultrasound in each gestational week between 20 and 24 were not same in the study which we investigate when the FAORs of GDM subjects were significantly different from those of NGT subjects. Inter-observer variability on the assessment via the ultrasonography was not evaluated because of the retrospective nature of this study. But there were no differences in the ultrasound scanners used, and all pregnant women scanned were randomly assigned to one of the three sonographers. The strengths of this study include a relatively large sample size with the same ethnicity and clinical management of all subjects according to the same protocol throughout the study period.

In summary, FAO was already present at 20–24 GW in the high risk older and/or obese GDM but not in the young and non-obese GDM subjects. Furthermore, FAO at 20–24 GW in GDM subjects was associated with higher odds ratios for FAO at the time of GDM diagnosis and LGA at birth, 10.15 and 5.57, respectively. These findings suggest that much earlier diagnosis and active interventions of GDM before or early in pregnancy might be necessary to prevent FAO ultimately resulting in metabolic abnormality even up to childhood and adolescence, especially in the high risk older and/or obese GDM women.

## Methods

### Subjects and data collection

We retrospectively reviewed the medical records of 7820 singleton pregnant women who were followed up at the outpatient clinic of CHA Gangnam Medical Center from January 1, 2012, to April 31, 2015. Among them, 6996 women who had fetal biometry data measured 20–24 GW and delivered at CHA Gangnam Medical Center were included in this study. The data on maternal height, body weight in pre-pregnancy and at the 50-g GCT, biochemical test, and fetal biometry were obtained from the medical records. All subjects included in this study were Asian. This study did not examine socioeconomic status. The data collection was approved by the Institutional Review Board (IRB) of CHA Gangnam Medical Center with a waiver of informed consent for the retrospective chart review (CHA Gangnam Medical Center-IRB No. GCI-18-10). All experiments were performed in accordance with relevant guidelines and regulations.

### Diagnosis of GDM

**Figure 1:**
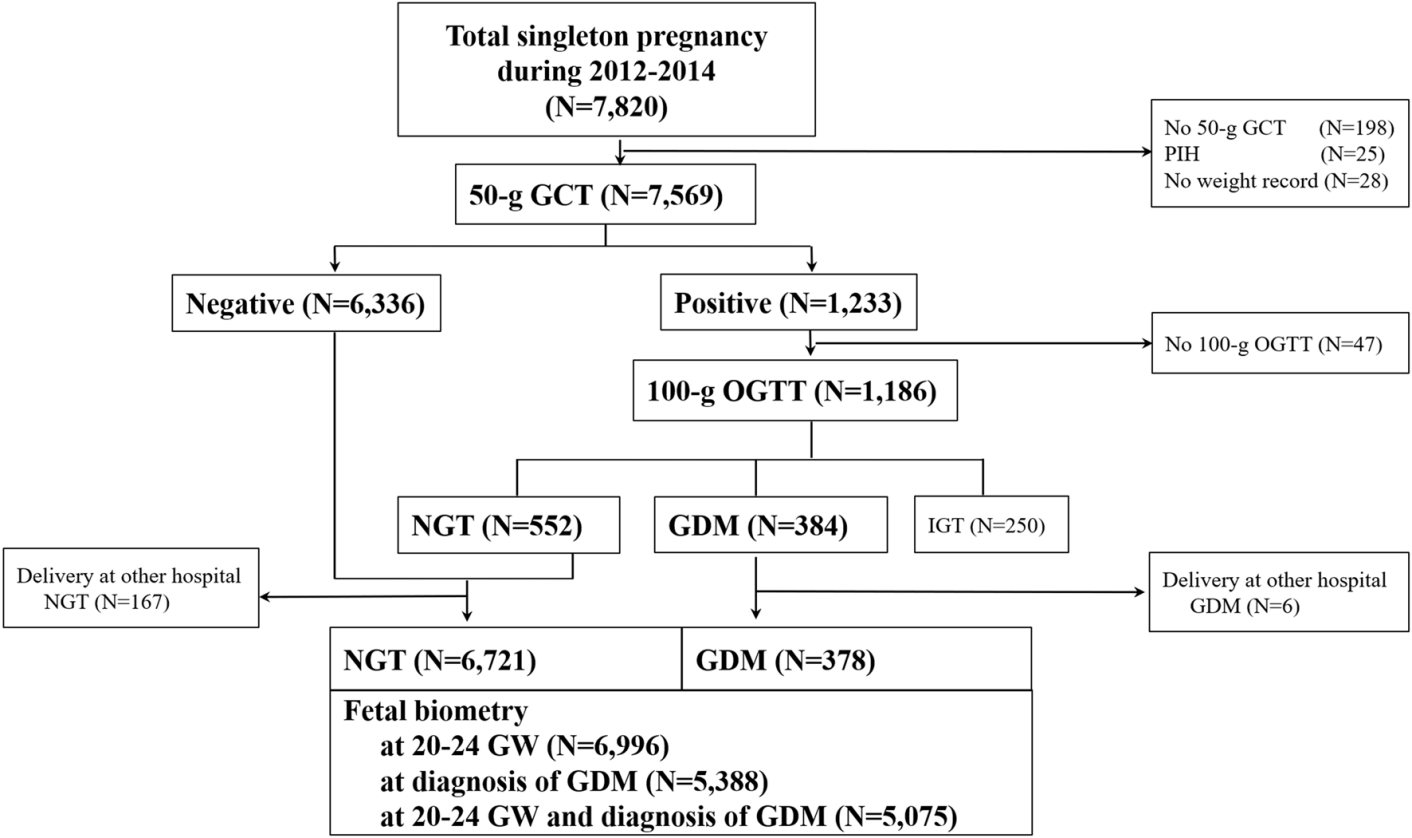
Study flow gram. *GCT* glucose challenge test, *PIH* pregnancy induced hypertension, *OGTT* oral glucose tolerance test, *NGT* normal glucose tolerance, *GDM* gestational diabetes mellitus, *IGT* impaired glucose tolerance, *GW* gestational week.

As described previously^17^, all pregnant women were universally recommended to undergo screening with a 50-g GCT irrespective of fasting at 24–28 GW and subsequent a 3-h 100-g OGTT with measurements of fasting insulin and HbA1c after more than an 8-h fasting if the 50-g GCT result was ≥ 140 mg/dL. The diagnosis of GDM and NGT depended on the Carpenter–Coustan criteria. Only one abnormal value on 100-g OGTT according to the Carpenter–Coustan criteria was diagnosed as impaired glucose tolerance (IGT). Among the total of 7569 subjects screened with a 50-g GCT, 1186 women with glucose ≥ 140 mg/dL on the 50-g GCT underwent a 100-g OGTT whereas 47 did not. Of these, 552 had NGT, 250 had impaired glucose tolerance, and 384 had GDM. From the 6888 NGT and 384 GDM subjects, 167 and 6 subjects delivered at other hospital respectively, and 82 and 21 subjects had no fetal biometry data measured at 20–24 GW, respectively. As a result, 6639 NGT and 357 GDM subjects were included in the study (Fig. 1). A total of 357 GDM subjects were divided into four study groups according to maternal age and pre-pregnancy BMI—group 1 (age < 35 years and BMI < 25 kg/m^2^ [n = 135]), group 2 (age < 35 years and BMI ≥ 25 kg/m^2^ [n = 20]), group 3 (age ≥ 35 years and BMI < 25 kg/m^2^ [n = 154]), and group 4 (age ≥ 35 years and BMI ≥ 25 kg/m^2^ [n = 48]).

### Fetal biometry

We collected fetal biometry data measured at 20–24 GW (n = 6996) as a routine anomaly scan and simultaneously with the 50-g GCT (n = 5388) at 24–28 GW. Among them, 5075 subjects had fetal biometry data measured at both times. Gestational dating was confirmed in 87% of these women by fetal ultrasonography performed prior to 14 GW. BPD, FL, and AC were measured three times via ultrasonography (GE Healthcare, USA) by one of the experienced 3 sonographers, and the mean values were converted to each estimated GA (i.e., GA-BPD, GA-FL, and GA-AC) according to the Japanese fetal growth chart^45,46^ (Fig. S1). But interobserver variability was not evaluated due to the retrospective nature of this study. We calculated 2 sets of FAORs as GA-AC/GA-LMP (actual GA measured by the last menstruation period) to correct for the variations in the ultrasound scan timing, and GA-AC/GA-BPD or GA-AC/GA-FL to detect overgrowth of the abdomen relative to the head and femur growth, respectively. The presence of FAO was defined as FAORs ≥ 90th percentile of the total subjects with fetal biometry (GA-AC/GA-LMP ≥ 1.076, GA-AC/GA-BPD ≥ 1.070, GA-AC/GA-FL ≥ 1.077 at 20–24 GW; GA-AC/GA-LMP ≥ 1.080, GA-AC/GA-BPD ≥ 1.071, GA-AC/GA-FL ≥ 1.069 at the time of 50-g GCT). Although bigger AC also could be due to enlarged liver or other causes, AC has been used as a proxy of increased fetal fat accretion. So, we defined fetal abdominal obesity as FAOR ≥ 90th percentile and use the term. The estimated fetal weight was calculated using the Shinozuka formula^47^. We defined LGA at birth as ≥ 90th percentile of gestational age matched birth weight according to the report of Committee of the Korean Society of Neonatology by Lee et al.^48^. Macrosomia was defined as infant birth weight ≥ 4 kg.

A diagnosis of GDM was made shortly after screening, usually within one week. Therefore, we use the phrase “at the time of GDM diagnosis” to describe the fetal biometry performed on the same day of the 50-g GCT. We also abbreviate this phrase to “at diagnosis.”

### Biochemical analysis

Plasma glucose was measured using the hexokinase method (Quailigentglu, Sekisui, Japan), and HbA1c was measured via high-performance liquid chromatography (G8 Elution Buffer, Tosoh, Tokyo, Japan). The plasma insulin concentration was determined via electrochemiluminescence immunoassay (ElecsysInsulin, Roche Diagnostics GmbH, Mannheim, Germany). Insulin resistance (homeostatic model assessment for insulin resistance [HOMA-IR]) and secretion (HOMA-β) were calculated by homeostasis model assessment^49^.

## Statistical analyses

Clinical and biochemical characteristics were reported as mean with standard deviation and proportion. Pregnancy outcomes such as primipara, cesarean delivery, primary cesarean delivery, LGA, and macrosomia were reported as proportions. Other pregnancy outcomes such as infant birth weight (g) and GA at delivery (weeks) were summarized as mean and standard deviation. Univariate associations of the characteristics or outcomes between NGT and total GDM or each of subgroups of GDM according to maternal age and pre-pregnancy BMI were investigated using two sample *t* test and test of two proportions. Pairwise associations among subgroups of GDM were investigated using post hoc analysis following one-way analysis of variance (ANOVA) using Tukey’s method for continuous variables. Where the assumptions of one-way ANOVA were not met, Kruskal–Wallis test followed by Dunn’s test as a post hoc analysis was conducted. For categorical variables, pairwise associations among GDM subgroups were investigated using test of two proportions for all possible pairs of subgroups.

Fetal biometry data and FAORs were described as mean and standard deviation and two sample *t* test was used for investigating univariate associations between NGT and total GDM or each subgroup of GDM. Assumptions of two sample *t* test were checked using Shapiro–Wilk test and Bartlett's test for the fetal biometry measures and FAORs.

Further, FAORs and EFW were summarized as mean and standard deviation and compared between NGT and GDM subjects according to each week of 20–24 GW using two sample *t* test.

For the subgroup analysis, within NGT and GDM, subjects with FAO at 20–24 GW and those without were compared for clinical and biochemical characteristics and pregnancy outcomes. Continuous variables were tested using two sample *t* test and categorical variables were compared using test of two proportions.

Odds ratio of having FAO at the time of GDM diagnosis, being LGA at birth, and macrosomia were estimated using logistic regression analysis models by NGT and GDM subject and by having FAO at 20–24 GW or not. NGT subjects with FAO at 20–24 GW and GDM subjects with and without FAO at 20–24 GW were compared to NGT subjects without FAO at 20–24 GW.

Correlations were assessed by Pearson’s correlation coefficient. All analyses were conducted using STATA version 15.1 (StataCorp LP, College Station, TX, USA). The level of significance for the analyses was 0.05.

### Subgroup analysis of GDM subjects according to maternal age and pre-pregnancy BMI

A total GDM subjects were divided into four study groups according to maternal age (35 years) and pre-pregnancy BMI (25 kg/m^2^). We compared the clinical and biochemical profiles and the fetal biometry data between GDM and NGT subjects. The GDM subgroup data were compared with each other and, also with those of the NGT subjects.

Logistic regression models were used to estimate the odds of FAO at 20–24 GW in total GDM or subgroup of GDM versus NGT subjects. Specific to GDM subgroup 2, an exact logistic regression model rather than a regular logistic regression model was implemented to reduce potential bias resulting from the lower number of participants in the group (n = 20).

### Subgroup analysis in the NGT and GDM subjects according to presence or absence of FAO at 20–24 GW

A total of 6996 subjects were divided into the following four study groups according to the presence or absence of FAO at 20–24 weeks of gestation: NGT without FAO (n = 5988), NGT with FAO (n = 651), GDM without FAO (n = 307), and GDM with FAO (n = 50). We compared the clinical and biochemical parameters of mothers and pregnancy outcomes between the study groups.

We also investigated odd ratios of exhibiting FAO at the time of GDM diagnosis, being LGA at birth, and macrosomia in the NGT subjects with FAO and GDM subjects with and without FAO at 20–24 GW relative to the NGT subjects without FAO at 20–24 GW.

### Subgroup analysis in the NGT and GDM subjects according to the GW at fetal biometry performed

Subjects were grouped according to GW at which fetal biometry was performed to investigate when GDM subjects begin to show higher FAORs than NGT subjects. The mean FAOR for each GW was compared between GDM and NGT subjects. Further, the odd ratios for FAO in GDM subjects were estimated in comparison with NGT subjects.

## Supplementary Information

Supplementary Information.

## Data Availability

Further information about data and resources will be provided upon request to the corresponding author.
