# Supplementary material for: Fetal abdominal overgrowth is already present at 20–24 gestational weeks prior to diagnosis of gestational diabetes mellitus
Source: Sci Rep. 2021 Dec 10;11:23821. doi: 10.1038/s41598-021-03145-7 (PMC8664824; doi:10.1038/s41598-021-03145-7)

Fetal abdominal Overgrowth is already present at 20-24 gestational weeks prior to diagnosis of gestational diabetes mellitus

Wonjin Kim, MD[1,2]*; Soo Kyung Park, MS[3]*; Yoo Lee Kim, MD, PhD[1]

[1]Division of Endocrinology and Metabolism, Department of Internal Medicine, Gangnam CHA Medical Center, CHA University School of Medicine, Seoul, Korea.

[2]Yonsei University College of Medicine, Seoul, Korea

[3]Department of Biostatistics and Data Science, University of Texas Health Science Center at Houston, Houston, Texas, 77030, USA

*: These two authors contributed equally to this work as co-first authors.

ORCID ID:

W Kim: 0000-0002-0689-4100

SK Park: 0000-0003-4708-630X

YL Kim: 0000-0002-9025-9774

Corresponding author:

Yoo Lee Kim, MD, PhD,

Division of Endocrinology and Metabolism, Department of Internal Medicine, Gangnam CHA Gangnam Medical Center, CHA University School of Medicine, 566, Nonhyeon-ro, Gangnam-gu, Seoul, 06135, Republic of Korea

Tel: +82-2-3468-3077; Fax: +82-2-3468-3565

E-mail: ylkim@cha.ac.kr, ylkim60@daum.net

Supplementary Table S1. Clinical and biochemical characteristics and pregnancy outcomes in NGT and GDM subjects with and without fetal biometry

	NGT (N=6,721)			GDM (N=378)		
	With fetal biometry data (n=6,639)	Without fetal biometry data (n=82)	p-value	With fetal biometry data (n=357)	Without fetal biometry data (n=21)	p-value
**Clinical and biochemical**						
Age (years)	33.2±3.8	32.0±3.7	<0.001	35.3±3.9	34.2±3.7	0.1935
Pre-pregnancy BMI (kg/m^2)	20.6±2.6	20.5±2.6	0.7393	22.3±3.4	21.8±4.1	0.5053
Weight gain (kg)						
Pre-pregnancy – At diagnosis	7.5±3.25	7.5±3.8	0.7455	7.8±3.6	8.0±3.5	0.8291
Pre-pregnancy – Near term	12.9±4.1	13.4±4.4	0.0296	11.4±4.6	12.7±4.6	0.2573
50-g GCT (mmol/L)	6.2±1.2	6.2±1.1	0.6712	9.2±1.4	8.9±1.4	0.4270
Fasting plasma glucose[a] (mmol/L)	4.5±0.4	4.5±0.3	0.9708	5.0±0.8	4.7±0.9	0.2130
HbA1c at diagnosis[a] (%)	5.0±0.3	5.0±0.3	0.8635	5.3±0.5	5.2±0.5	0.4017
Fasting insulin[a] (pmol/L)	7.9±4.0	11.4±10.6	0.2429	9.9±4.9	9.6±5.5	0.7593
Insulin treatment (%)	-	-		10.9	4.8	0.3723
**Pregnancy outcomes**						
Primipara (%)	67.4	65.7	0.4910	66.7	66.7	1.0000
Cesarean delivery (%)	36.1	35.7	0.8792	50.1	47.1	0.8037

Primary cesarean delivery (%)	21.9	15.3	0.0024	31.1	14.3	0.1029
Infant birth weight (gm)	3195.1±419.4	3240.4±445.9	0.0637	3224.4±516.5	3261.2±396.0	0.7726
GA at delivery (weeks)	39.0±1.5	39.0±1.68	0.3912	38.4±1.8	38.7±0.9	0.4671
LGA (%)	5.1	8.0	0.0409	13.6	11.8	0.8247
Macrosomia[b] (%)	1.9	2.9	0.3127	4.7	5.9	0.8310

NGT, normal glucose tolerance; GDM, gestational diabetes mellitus; BMI, body mass index; GCT, glucose challenge test; HbA1c, glycosylated hemoglobin; GA, gestational age; LGA, large for gestational age.

[a] Column Ns for the variable are different from the Ns presented in the table, [b] Infant birth weight ≥ 4kg

Supplementary Table S2. Odds ratios of FAO by each FAOR at 20-24 GW in NGT, total GDM, and GDM subgroups

	Fetal Abdominal Obesity Odds Ratio (95% CI)		
	GA-AC/GA-LMP[b] ≥90th	GA-AC/GA-BPD ≥90th	GA-AC/GA-FL ≥90th
NGT	1 (ref.)	1 (ref.)	1 (ref.)
Total GDM	1.496[a] (1.097, 2.038)	1.088 (0.770, 1.538)	1.589[a] (1.171, 2.156)
GDM Group 1	0.978 (0.549, 1.743)	0.973 (0.546, 1.734)	1.253 (0.739, 2.126)
GDM Group 2	1.620 (0.474, 5.544)	3.915[a] (1.500, 10.223)	2.331 (0.777, 6.992)
GDM Group 3	2.041[a] (1.344, 3.098)	0.986 (0.575, 1.690)	1.637[a] (1.043, 2.568)
GDM Group 4	1.312 (0.556, 3.098)	0.831 (0 297, 2.319)	2.151[a] (1.037, 4.461)

FAO, fetal abdominal obesity; FAOR, fetal abdominal overgrowth ratio; GW, gestational weeks; GA-AC, estimated gestational age by abdominal circumference; GA-LMP, gestational age by last menstruation period; GA-BPD, estimated gestational age by biparietal diameter; GA-FL, estimated gestational age by femur length; NGT, normal glucose tolerance; GDM, gestational diabetes mellitus.

[a]$p < 0.05$, [b]Gestational age by LMP at 20-24 GW, [c]Fetal abdominal overgrowth defined as fetal abdominal overgrowth ratio (FAOR) ≥ 90th were GA-AC/GA-LMP ≥1.076, GA-AC/GA-BPD ≥1.070, and GA-AC/GA-FL ≥1.077

Supplementary Table S3. Odds ratios of FAO by FAOR of GA-AC/GA-LMP ≥ 90th according to GW at which fetal biometry was performed.

	Odd ratios of FAO by FAOR ≥ 90th	
	NGT	GDM
20-21 GW	1 (ref.)	1.624 (0.581, 4.536)
21-22 GW	1 (ref.)	1.731 (1.081, 2.772)
22-23 GW	1 (ref.)	1.474 (0.923, 2.353)
23-24 GW	1 (ref.)	0.999 (0.404, 2.474)

FAO, fetal abdominal obesity; FAOR, fetal abdominal overgrowth ratio; GA-AC, estimated gestational age by abdominal circumference; GA-LMP, gestational age by last menstruation period; GW, gestational week; NGT, normal glucose tolerance; GDM, gestational diabetes mellitus.

Supplementary Table S4. Correlation of FAORs measured at 20-24 GW with FAORs measured at the time of diagnosis of GDM

FAORs	Variables	Correlation Coefficient	p-value
GA-AC / GA-LMP<br>- at 20-24GW	GA-AC / GA-LMP<br>- at diagnosis	0.4348	<0.0001
GA-AC / GA-BPD<br>- at 20-24 GW	GA-AC / GA-BPD<br>- at diagnosis	0.3054	<0.0001
GA-AC / GA-FL<br>- at 20-24 GW	GA-AC / GA-FL<br>- at diagnosis	0.2839	<0.0001

FAOR, fetal abdominal overgrowth ratio; GW, gestational week; GDM, gestational diabetes mellitus; GA-AC, estimated gestational age by abdominal circumference; GA-LMP, gestational age by last menstruation period; GA-BPD, estimated gestational age by biparietal diameter; GA-FL, estimated gestational age by femur length.

Fig. S1. Measurement of fetal biparietal diameter, femur length, and abdominal circumference by ultrasonography and conversion to estimated gestational age

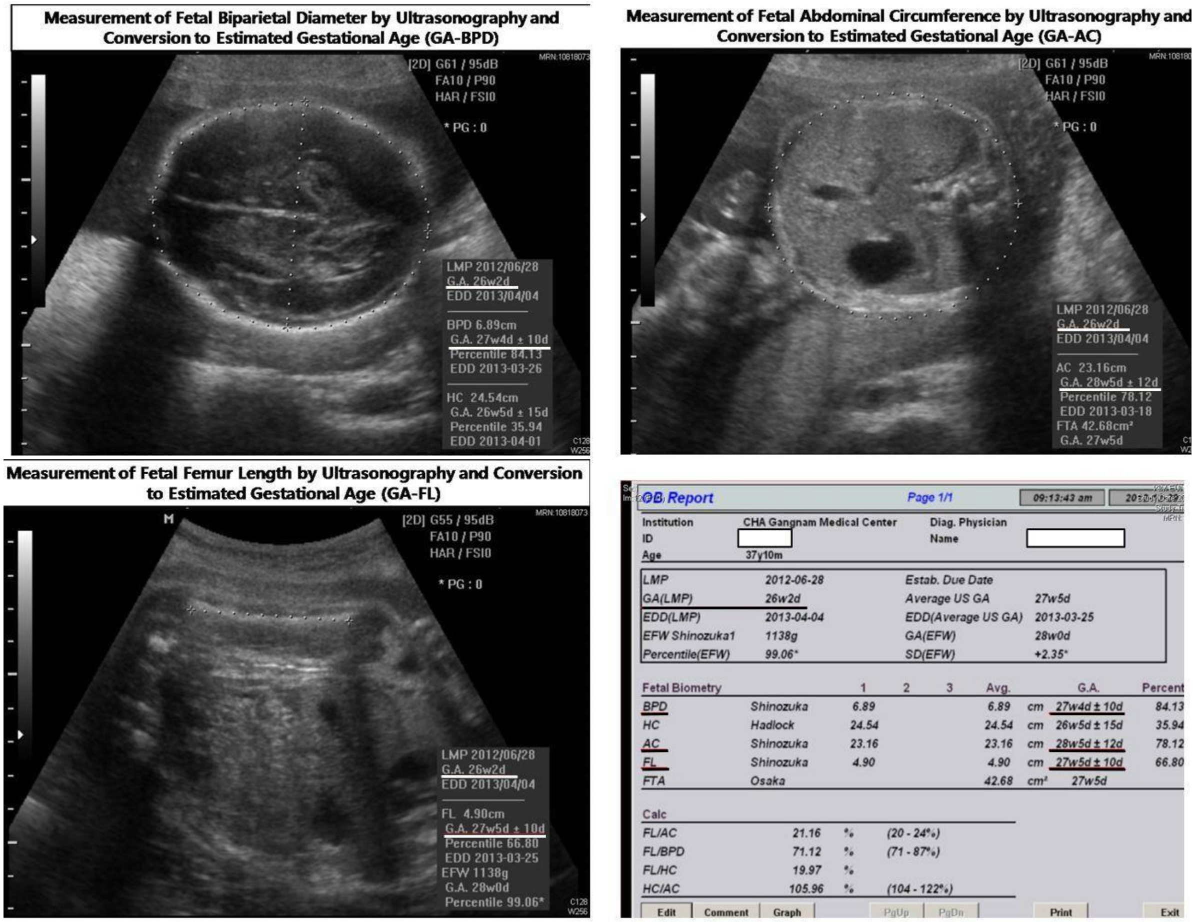

Supplement: Supplementary file 1 — Supplementary Information. [file 41598_2021_3145_MOESM1_ESM.pdf]
